# Levels of Physical Activity in Children and Adolescents with Type 1 Diabetes in Relation to the Healthy Comparators and to the Method of Insulin Therapy Used

**DOI:** 10.3390/ijerph16183498

**Published:** 2019-09-19

**Authors:** Ewelina Czenczek-Lewandowska, Justyna Leszczak, Joanna Baran, Aneta Weres, Justyna Wyszyńska, Bogumił Lewandowski, Mariusz Dąbrowski, Artur Mazur

**Affiliations:** 1 Faculty of Medicine, University of Rzeszów, 35-959 Rzeszów, Poland; leszczakjustyna.ur@gmail.com (J.L.); joannabaran.ur@gmail.com (J.B.); anetaweres.ur@gmail.com (A.W.); boglewandowski@wp.pl (B.L.); mariusz.dabrowski58@gmail.com (M.D.); drmazur@poczta.onet.pl (A.M.); 2 Clinical Regional Hospital No. 2 in Rzeszów, Lwowska Street 60, 35-301 Rzeszów, Poland

**Keywords:** type 1 diabetes, physical activity, children, insulin therapy

## Abstract

Given the fact that physical exertion leads to blood glucose fluctuations, type one diabetes mellitus (T1D) may potentially constitute a barrier for obtaining a sufficient amount of exercise. The main purpose of the study was to compare the level of physical activity between children with T1D (*n* = 215) and healthy controls (*n* = 115) and to assess the physical activity of the study group in relation to the applied method of insulin therapy, i.e., the use of insulin pen vs. insulin pump. The level of physical activity was assessed with a hip-worn tri-axial accelerometer (ActiGraph GT3X+) used by the subjects for an uninterrupted period of seven days. Children with T1D had significantly lower median values of total time of moderate (213.3 vs. 272.1 min), vigorous (135.3 vs. 19.6 min) and moderate-to-vigorous (347.4 vs. 467.4 min) physical activity compared to healthy peers respectively, (*p* < 0.001) in all cases. In addition, the total median number of steps was significantly lower (53,631 vs. 67,542 steps), (*p* < 0.001). The method of insulin therapy was not associated with significant differences in physical activity level (*p* > 0.001). The level of physical activity in children and adolescents with T1D is lower than in their healthy peers and does not depend on the insulin therapy method.

## 1. Introduction

Type one diabetes (T1D) is one of the most common diseases occurring during developmental age and it affects a growing number of young children worldwide [1,2]. The number of new cases was approximately 86,000 in 2014 and 132,600 in 2016, and in 2017 the total number of children with T1D exceeded 1.1 million [3,4,5]. The condition is particularly challenging for children and adolescents as they find it difficult to adopt the necessary discipline and the changes in lifestyle needed to control their diabetes. The disease is associated with a number of duties, as those affected must regularly perform blood glucose tests, follow a recommended diet, and apply functional insulin therapy (FIT) that is administered subcutaneously either by multiple daily injections (MDI) or by continuous subcutaneous insulin infusion (CSII). Implementation of all those duties may induce problems for children, especially the youngest ones [6,7].

T1D in children and adolescents may adversely affect relations with peers, which can lead to difficulties at school, impairment of sleep, mood swings, and disturb the daily functioning of the family unit [8].

Highly variable glucose responses to physical activity may present a major challenge and thus the need for management of food intake and insulin dosing. To prevent hypoglycemia during aerobic exercise lasting more than 30 min, additional carbohydrate intake and/or reductions in basal and/or bolus insulin dose are required [9]. Very intensive exercise can cause hyperglycemia, especially when blood glucose levels are elevated before exercise. Prevention of post-exercise hyperglycemia is based on insulin correction based on an individual’s insulin correction factor (100% or 150%) [10]. Measuring blood glucose levels before physical activity is mandatory and it should range between 90 and 250 mg/dL. Exercise-related hypoglycemia or hyperglycemia and the ability to counteract it means that for children with T1D, physical activity often poses a considerable challenge, and there are some risks that can be neglected [11]. This fact is of tremendous importance since in addition to necessary insulin therapy and customized diet, physical activity is an indispensable element of diabetes control that favorably affects a patient’s condition and contributes to the positive effects of therapy [8,12].

Given the fact that children who are naturally more active are also happier, healthier and more eager to face new challenges, the issues related to physical activity in chronic conditions seem to be of critical importance [13,14]. The main objective of the present study was to analyze differences in the level of physical activity between children and adolescents with T1D and healthy controls. The secondary aim of the study was to analyze whether the method of insulin treatment: MDI vs. CSII, is associated with different levels of physical activity.

## 2. Materials and Methods

### 2.1. Participants

The study was conducted in a group of 451 children of 6 to 18 years of age, who agreed to wear for 7 days an accelerometer, which is a small device that allows objective measurement of physical activity. The children were examined in a diabetes outpatient clinic in a clinical hospital where they undergo follow-up visits. Participation in the study was voluntary and anonymous. The study group consisted of 286 children with T1D fulfilling the inclusion criteria: Ages from 6 to 18 years, T1D diagnosed at least one year before the start of the study, informed consent signed by the parent and adolescents aged over 16 years, or confirmed orally by the children below 16 years, a record of physical activity using an accelerometer for a duration of >500 min for a minimum of four days out of the seven days of the study [15].

The control group consisted of 165 healthy children and adolescents fulfilling the inclusion criteria with the exception of having T1D. The control group was recruited in randomly selected primary and high schools with the standard number of PE (physical education) hours, without sport profile. All the parents or legal guardians, as well as children and adolescents participating in the study were instructed on the use of the accelerometer.

**Figure 1:**
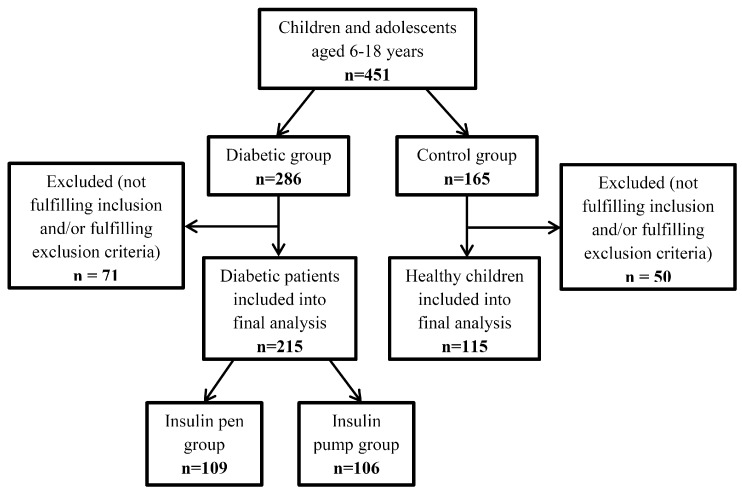
Flow chart of study participant selection.

The exclusion criteria consisted of: Having type two or other type of diabetes (MODY (Maturity Onset Diabetes of the Young), diabetes associated with endocrine disorders, etc.), suffering from other metabolic disorders, having microvascular complications: Retinopathy, nephropathy, and neuropathy; or macrovascular complications: Cardiovascular disease, cerebrovascular accidents, and peripheral vascular disease, or having any other medical conditions during the study. Other contraindications were related to the timing of the study, such as extremely bad weather, holidays or the summer break, when the children’s physical activity could significantly differ from regular daily routines [16]. After a short interview when returning the accelerometer, the children and adolescents who failed to meet the requirements of the study were excluded. The most common reasons for exclusion were too short time of wearing the accelerometer because of a failure to follow instructions or the child’s illness during the examination period. Finally, 215 children and adolescents with T1D and 115 healthy comparators were included in the analysis (Figure 1).

According to demographic data, in the area where the research was carried out, 4800 children have diabetes. The sample size was calculated based on the total number of patients with T1D between 6 to 18 years of age from the region of the country where the study was conducted (*n* = 500). This means that the incidence of T1D in the region is 10.5%. The size of the required sample was calculated, taking into account a 95% confidence level and the level of significance was considered as *p* < 0.05. It was calculated that the minimum sample size should be 141 children.

### 2.2. Procedures

Assessment of physical activity was performed using a tri-axial accelerometer in a GT3X-BT Monitor (ActiGraph, Pensacola, FL, USA). The device detects body movement acceleration in three planes, and it is the most commonly used measure for child subjects. Due to the device’s piezoelectric sensor that transforms an analogue signal into a digital one, it enables reliable and accurate monitoring of physical activity [17].

In the children with T1D, medical history was collected in accordance with the Pediatric Care Summary Report, including information about the patient, the course of the disease and the applied therapy. The report was completed by the lead researcher. At the next stage, the accelerometer was installed and the parent as well as the study participants were instructed the use of the device. With the healthy controls, the same procedure was applied; however, in this case, a specially designed questionnaire was used instead of the Pediatric Care Summary Report.

Each participant in the study was instructed to attach an accelerometer to the hip-waist area with a flexible strap and to wear it for 12 h daily for 7 days excluding night time and during activities performed in contact with water (bathing, swimming). Any 30-min long periods of consecutive zeros in the read-outs were classified as non-wear time or sleep time and were excluded from the analyses. The measurements were performed with a sampling rate of 30 Hz and analyses applied in 10 s epochs. The parameters of physical activity were computed using the dedicated Actilife software (Actilife software, version 6.8.3, ActiGraph, Pensacola, FL, USA). The data presented the durations of sedentary activities <100 count per minutes (cpm), light physical activity (LPA), 101–2295 cpm, moderate physical activity (MPA), 2296–4011 cpm, and vigorous physical activity (VPA), >4012 cpm. These values were calculated in time units (min/h) according to the algorithm Freedson Children (2005) [18] and as per cent values according to the algorithm Evenson Children (2008) [19]. Additionally, identified rates represented moderate to vigorous physical activity (MVPA), >2296 cpm, total MVPA (min), MVPA%, mean MVPA/day, as well as total number of steps, mean number of steps per day and minute, according to the algorithm Freedson Children (2005). The minimum duration of physical activity recording was defined as >500 min, to be registered during a minimum of four days out of the seven days of the study.

### 2.3. Reference Norms of Physical Activity Adopted for the Study Participants

The MVPA reference value was based on the recommendations of the World Health Organization (WHO) of 2010, which determine the minimum desired time of this activity for children and adolescents as ≥60 min a day [20]. The reference value for the VPA coefficient was taken from the guidelines of the US Department of Health and Human Services published in 2010. According to these guidelines, VPA should last no less than 20 min a day, at least 3 times a week [21]. The number of steps referred to the Tudor Locke recommendations of 2011, according to which this number should exceed 13,000 steps a day for boys and 11,000 steps a day for girls aged 6–11 years, while for adolescents between 12 and 19 years, it should be over 10,000 steps a day [22].

### 2.4. Statistical Analysis

Statistical analysis was performed using the Statistica 10.0 software (StatSoft Polska Sp. z o.o, Cracow, Poland) based on the data records from a minimum of four valid days of the study. Continuous variables were presented as mean and median values, and due to the non-parametric distribution they were compared using a Mann–Whitney U-Test. Nominal variables were presented as number and percentage, and they were compared using a χ^2^ test. In the first step we compared results obtained in study group as a whole with healthy controls. Then, we compared results from the study group divided according to the method of treatment. The level of statistical significance was adopted at *p* < 0.05.

### 2.5. Ethics

The patients signed an informed consent to participate in the study. The study was approved by the Bioethics Committee at the Medical Department of the University of Rzeszów, decision on 17/12/2015, and it was conducted in accordance with ethical standards laid down in an appropriate version of the Declaration of Helsinki (as revised in Brazil 2013).

## 3. Results

A total of 330 children and adolescents aged from 6 to 18 years were included in the final analysis. In this number there were 215 (65.2%) participants with T1D and 115 (34.8%) healthy controls. The age and body weight of participants in the study and control groups were not significantly different (mean age = 12.61 years ± 3.26 SD vs. 11.98 years ± 2.76 SD; BMI (body mass index) = 23.87 kg/m^2^ ± 4.52 SD vs. 22.55 kg/m^2^ ± 5.47 SD). The ratio of girls and boys was 3:2 in the diabetes group, 1:1 in the control group, 1:1 in MDI and 3:2 in CSII groups.

We revealed significant differences between individuals with T1D and control groups in all of the analyzed variables, with the exception of sedentary activities, time spent in LPA and percentage of time spent in LPA (Table 1).

Next, the patients with T1D were divided based on insulin therapy into an MDI group of 109 children (50.7%) treated with insulin pens and a CSII group of 106 children (49.3%) treated with insulin pumps. The mean duration of insulin therapy was 3.94 years ± 3.13 SD in the diabetes group, 3.57 ± 3.08 SD in the MDI group and 4.32 ± 3.15 SD in the CSII group. Mean HbA1C (glycated haemoglobin) in the year of the study was 7.38% (57 mmol/mol) ± 1.12 SD in the diabetes group, 7.37% (57 mmol/mol) ± 1.20 SD in the MDI group and 7.40% (57 mmol/mol) ± 1.03 SD in the CSII group. When we performed a comparison of physical activity between the two groups, we did not find any significant differences (Table 2).

In the analysis, the fulfilment of the MVPA, VPA recommendations and the number of steps were taken into account. Among the subjects with diabetes, significantly fewer participants fulfilled the recommended level of physical activity compared to the control group. No significant differences between the MDI and CSII subgroups were found (Table 3).

It was shown that the diabetic girls obtained significantly higher results compared to the healthy girls in the case of parameters (i.e., vigorous (VPA) (min), % sedentary and % vigorous (VPA)). Parameters moderate (min), % light, % moderate, total mvpa, % MVPA, mean MVPA/per day, total number of steps/study period, mean number of steps/per day and mean number of steps/per minute were higher among girls from the control group. It was shown that diabetic boys obtained significantly higher results compared to boys from the control group in the case of parameters such as total MVPA, % MVPA, mean MVPA/per day. Parameters MODERATE (min), total number of steps/study period, mean number of steps/per day and mean number of steps/per minute were higher among boys from the control group (Table 4).

The statistical analysis showed statistically significant differences between girls and boys with T1D in four aspects (i.e., % SEDENTARY (*p* = 0.014), % LPA (*p* = 0.036), % MVPA (*p* = 0.038) and mean MVPA/ day (*p* = 0.041)). The value of the % SEDENTARY parameter was significantly higher in the girls’ group, while the other parameters were higher in the boys group. The presence of statistically significant differences between the results obtained by girls and boys from the control group (*p* > 0.05) was not confirmed (Table 5).

It was shown that children 6–12 years from the study group obtained significantly higher results compared to peers from the control group in the case of parameters (i.e., vigorous (VPA) (min) and % Sedentary). Parameters moderate (min), % moderate, % vigorous (VPA), total MVPA, % MVPA, mean MVPA/per day, total number of steps/study period, mean number of steps/per day and mean number of steps/per minute higher among the control group. It was shown that children aged 13–18 from the control group obtained significantly higher results compared to peers from the study group in the case of total parameters (Table 6).

The analysis showed statistically significant differences between children with T1D aged 6–12 and 13–18 years in three aspects (i.e., LPA (min) (*p* < 0.001), % SEDENTARY (*p* < 0.001) and % LPA (*p* < 0.001)). The value of the % SEDENTARY parameter was significantly higher in children aged 13–18, while the other two parameters were higher among children aged 6–12. Statistically significant differences in all parameters were found in children aged 6–12 and 13–18 from the control group. The value of the SEDENTARY time (min) and % SEDENTARY were significantly higher in children aged 13–18 years, while the other parameters were higher among children aged 6–12 years (Table 7).

Statistically significant differences in the study and control group among girls aged 6–12 years were found for the marked parameters. The values obtained by the children from the control group were always higher. Statistically significant differences in the study and control group among girls aged 13–18 years were found for the marked parameters. The values obtained by the subjects from the control group were always higher. Statistically significant differences in the study and control group among boys aged 6–12 years were found for the marked parameters. In the study group, the parameters vigorous, % sedentary, % vigorous and total MVPA were higher, while in the control group% MVPA, total number of steps/study period, mean number of steps / per day and mean number of steps/per minute. In the 13–18 age group, no statistically significant differences were found in boys (Table 8).

It was found that, in the study group, age affected the values of the following parameters: SEDENTARY, LIGHT, % SEDENTARY and % LIGHT. The values of the parameters sedentary and % sedentary were higher in the group of older subjects (13–18 years), and the values of the parameters light and % light were higher in the group of the younger subjects (6–12 years). The findings showed no effects of the factor of sex and no concurrent impact of the subjects’ sex and age in the results. In the control group, effects of age were observed in the values of all the parameters but not in the category VIGOROUS. The values of the parameters SEDENTARY and % SEDENTARY were higher in the group of older subjects (13–18 years), while the values of all the remaining parameters were higher in the group of younger subjects (6–12 years). No concurrent effects of sex and age were found in any of the parameters; likewise, the factor of sex alone did not impact the results (Table 9).

## 4. Discussion

Regular physical activity is considered to be a beneficial and necessary part of treatment of children and adolescents with T1D. It is one of the most important ways to effectively control diabetes, decrease insulin demand and reduce the risk of chronic complications, such as cardiovascular disease and hypertension, which show a 10-fold more frequent occurrence in patients with T1D compared to the healthy population [23,24]. Physical activity has a beneficial impact on lipid profile, blood pressure and endothelial function, and also improves mental well-being, which is very important, especially for teenagers [25]. Data from recent studies indicate a significant association of self-reported MVPA with better metabolic control and lower HbA1c in children and adolescents, which was also confirmed in a study with the use of an accelerometer [26]. Lack of physical activity is strongly associated with weight gain and obesity in patients with T1D, which leads to deterioration of metabolic control [14].

Because exercise leads to fluctuations in blood glucose level, it may be difficult for children and adolescents with T1D to undertake appropriate levels of physical activity in their everyday life [27]. An effective solution can be found in the use of new technologies such as continuous glucose monitoring (CGM), either from real-time use (rtCGM) or intermittently viewed (iCGM), which offer opportunities to improve self-management, allow observation of the trends in their glycemic control, as well as prevent hypoglycemia [28]. Fear of severe hypoglycemia is still the most important barrier for children and adolescents and it can be more troublesome for them than for adults with T1D [23,29]. This has a considerable effect on the daily habits of young patients with T1D, leading to decreased involvement in physical activity in their daily life. Recent studies indicate that for many reasons, the majority of children and adolescents with T1D do not achieve the recommended level of physical activity [30]. In studies by Maggio et al. and Trigona et al., between 35–39% of the study group and 57–60% of the controls met the recommended 60-min duration of MVPA [31,32,33]. Differences between the groups can already be observed in children with T1D below seven years of age, who achieved a daily value of MVPA 16 min lower than that of their healthy peers [34]. Insufficient levels of physical activity in children with T1D is a worldwide problem, yet the relevant rates are sometimes comparable to those observed in healthy children. Most studies report that the recommended daily MVPA of 60 min is not met by subjects representing either population [35].

The importance of performing a sufficient number of steps per day from the viewpoint of T1D control has been discussed by many authors. It has been established that an insufficient number of daily steps is strongly associated with premature symptoms of atherosclerosis. Every additional 1000 steps per day contributes to a reduced risk of cardiovascular diseases, which is extremely valuable in T1D management [36]. In the present study, only 26% of the study group and 44% of the healthy controls met the recommended level of 10,000 steps per day, which is assessed as unsatisfactory. For comparison, children with T1D in the SEARCH for Diabetes in Youth Case-Control Project, were found to have performed lower scores, and the lowest results were achieved by children with type two diabetes and healthy children [37]. It could be expected that due to the greater flexibility of insulin pumps, children and adolescents with T1D treated with this method will be more physically active compared to those treated with insulin pens. However, the present study did not confirm these expectations of insufficient levels of physical activity in children and adolescents with T1D. In addition, in another study, the type of therapy did not play a significant role [17,38].

At the same time, an increased time spent in sedentary behaviors and inactivity has been regularly observed [39]. In the present study, sedentary behaviors in children and adolescents in the study group accounted for a large majority of their time. These results are similar to those reported by other authors, amounting to 73% of daily time in the diabetes group and 70% in the control group. Most children watch TV for more than two hours a day, and this factor is closely related to being overweight or obese, which is more and more common in children and adolescents with T1D [40]. The related guidelines emphasize the need to decrease the duration of typical sedentary behaviors (sitting and lying down) in favor of light physical activity, such as walking, the latter involving a greater number of steps. Satisfactory MVPA rates are not automatically equivalent to beneficial health effects if a child does not have good quality sleep or spends too much time on sedentary activities [41,42].

A review of the evidence related to physical activity in children and adolescents with T1D indicates the necessity to introduce comprehensive preventive measures to promote increased physical activity and to reduce sedentary behaviors in this group of patients [22,29]. It requires the application of glucose self-monitoring during physical exercise, and the ability to balance physical exertion with proper diet and insulin intake.

Children and adolescents with T1D, irrespective of the therapy used, can be as active as their healthy peers if they receive adequate support from their therapists and schoolteachers [43]. It is necessary to introduce educational programs in schools for children, parents and teachers, who should broaden their knowledge about physical exercise in diabetes and how to prevent dangerous fluctuations in glucose. Use of CGM, rtCGM and iCGM should be widespread as they prevent the fear of exercise-induced hypoglycemia by constant observation of blood glucose trends which allow children to feel safer during physical activity [11]. Although preventive programs should be addressed in particular to children with chronic diseases, including T1D, it is also necessary to be aware that the problem of insufficient physical activity is also observed among children who are not affected by medical conditions [44]. Well-trained children and adolescents with well-controlled diabetes are able to perform high-intensity physical activity, just like their healthy peers. Given the above, practical strategies to improve engagement towards a more active lifestyle should be offered to all patients [45]. The latest guidelines published in 2018 by the International Society for Pediatric and Adolescent Diabetes (ISPAD) point out that children (aged 5–11 years) and adolescents (aged 12–17 years) should perform physical activity for a minimum of 60 min per day, including vigorous physical activity for a minimum of 20 min, and they should minimize sedentary time each day [12]. Likewise, the recommendations of Diabetes Poland (the Polish Diabetes Association) published in 2018 specify that in order to achieve the most effective diabetes control, physical activity should be undertaken each day, or for a minimum of two to three days per week. For the best effects, the proper activity should be preceded by five to ten min of introductory warm-up, and it should be followed with calming activities, e.g., relaxing or stretching exercises [46,47]. Physical inactivity affects an increasing number of people in the world of every age, including children in early childhood, hence it is considered a global public health problem.

## 5. Limitation

The limitation of the research is the small number of participants. The next planned tests will be carried out on a larger population. A valuable supplement to the presented research would be the use of training in the examined people, in accordance with the above guidelines of the diabetes association.

## 6. Conclusions

The level of physical activity in children and adolescents with T1D is lower than in their healthy peers and does not depend on the insulin therapy method applied.

## Figures and Tables

**Table 1:** Comparison of physical activity rates in the diabetes and the control groups.

Physical Activity Rates/Data from 7-Day Study	Diabetes Group (*n* = 215)					Control Group (*n* = 115)						
	Mean	−95.0% CI	95.0% CI	Me	SD	Mean	−95.0% CI	95.0% CI	Me	SD	Z Score	*p* Value
SEDENTARY [min]	3678.1	3523.6	3832.7	3711.1	±1149.7	3586.9	3422.3	3751.4	3575.3	±891.0	0.76	0.447
LIGHT (LPA) [min]	1046.1	995.6	1096.7	1010.7	±375.9	1055.6	997.7	1113.4	1073.8	±313.1	−0.74	0.460
MODERATE [min]	254.8	233.5	276.1	213.3	±158.7	268.3	252.9	283.8	272.1	±83.7	−**3.75**	<**0.001** ***
VIGOROUS [min]	232.2	197.4	267.0	135.3	±259.0	209.2	190.2	228.1	196.6	±102.6	−**3.99**	<**0.001** ***
% SEDENTARY	70.3	68.6	71.9	73.4	±12.3	69.6	68.1	71.1	69.8	±8.1	**2.25**	**0.024** *
% LIGHT (LPA)	20.3	19.4	21.1	19.5	±6.3	20.9	19.8	22.0	20.5	±5.9	−1.13	0.260
% MODERATE (MPA)	5.0	4.6	5.3	4.1	±2.9	5.4	5.0	5.7	5.5	±1.8	−**3.80**	<**0.001** ***
% VIGOROUS (VPA)	4.5	3.8	5.2	2.5	±4.9	4.1	3.8	4.5	3.7	±2.0	−**4.27**	<**0.001** ***
Total moderate to vigorous physical activity (MVPA)	487.0	432.3	541.7	347.4	±406.9	477.5	448.1	506.8	467.4	±158.9	−**4.49**	<**0.001** ***
% MVPA	9.5	8.4	10.5	6.6	±7.6	9.5	8.9	10.1	9.0	±3.3	−**4.62**	<**0.001** ***
Mean MVPA/day	76.2	67.9	84.4	52.0	±61.5	76.4	71.7	81.1	72.4	±25.6	−**5.05**	<**0.001** ***
Total number of steps/study period	56,856	54,022.6	59,690.0	53,631	±21,079.7	66,792	63,792.9	69,790.3	67,542	±16,233.0	−**4.87**	<**0.001** ***
Mean number of steps/day	8925	8504.9	9345.4	8307	±3126.0	10,717	10,211.4	11,222.5	10,616	±2736.5	−**5.65**	<**0.001** ***
Mean number of steps/minute	11.1	10.6	11.7	10.6	±4.0	13.3	12.6	14.0	13.3	±3.6	−**5.21**	<**0.001** ***

*n*—Number of participants; Me—median; Z score = result of Mann–Whitney U-Test; CI—confidence interval; SD—standard deviation; *p* value—probability level; *** *p* < 0.001, * *p* < 0.05; bold values—statistically significant.

**Table 2:** Comparison of physical activity rates in the multiple daily injections (MDI) and continuous subcutaneous insulin infusion (CSII) groups.

Physical Activity Rates/Data from 7-Day Study	MDI Group (*n* = 109)					CSII Group (*n* = 106)						
	Mean	−95.0% CI	95.0% CI	Me	SD	Mean	−95.0% CI	95.0% CI	Me	SD	Z Score	*p* Value
SEDENTARY [min]	3550.5	3325.1	3775.8	3473.7	1186.7	3809.4	3597.4	4021.3	3786.6	1100.5	−1.71	0.087
LIGHT (LPA) [min]	1053.4	980.1	1126.7	1006.0	386.1	1038.7	968.0	1109.3	1027.8	366.8	0.01	0.99
MODERATE [min]	258.2	227.5	288.9	213.6	161.5	251.3	221.2	281.5	211.4	156.8	0.25	0.799
VIGOROUS [min]	242.0	191.3	292.7	137.1	267.0	222.2	173.7	270.6	129.9	251.4	0.67	0.502
% SEDENTARY	69.0	66.4	71.6	72.9	13.5	71.6	69.5	73.7	74.3	10.8	−1.05	0.294
% LIGHT (LPA)	21.0	19.6	22.3	19.9	6.9	19.6	18.5	20.6	19.3	5.5	1.2	0.229
% MODERATE (MPA)	5.2	4.6	5.8	4.2	3.2	4.7	4.2	5.3	4.1	2.7	0.71	0.477
% VIGOROUS (VPA)	4.9	3.9	5.9	2.7	5.4	4.1	3.3	5.0	2.5	4.4	0.87	0.382
TOTAL MVPA	500.2	420.9	579.5	362.2	417.6	473.5	397.0	550.0	337.4	397.1	0.61	0.541
% MVPA	10.0	8.5	11.6	6.8	8.3	8.8	7.5	10.2	6.6	6.8	0.69	0.488
Mean MVPA/day	80.2	67.6	92.8	54.6	66.5	72.0	61.2	82.7	50.9	55.8	0.54	0.591
Total number of steps/study period	56,711.9	52,596.6	60,827.1	53,631.0	21,675.3	57,004.8	53,046.9	60,962.7	53,611.0	20,551.2	−0.28	0.781
Mean number of steps/day	9059.1	8410.7	9707.6	8604.4	3415.3	8787.4	8246.7	9328.0	8184.2	2807.4	0.03	0.976
Mean number of steps/minute	11.4	10.6	12.2	10.9	4.2	10.9	10.2	11.6	10.4	3.8	0.71	0.475

*n*—Number of participants; Me—median; Z score = result of Mann–Whitney U-Test; CI—confidence interval; SD—standard deviation; *p* value—probability level.

**Table 3:** Fulfillment of recommended MVPA, VPA and number of steps norms in the study groups.

	Diabetes Group *n* = 215		Control Group *n* = 115		*p* Value	MDI Group *n* = 109		CSII Group *n* = 106		*p* Value
	*N*	%	*n*	%		*N*	%	*n*	%	
MVPA										
≥60 min a day	83	38.6%	87	75.7%	<**0.001** ***	44	40.4%	39	36.8%	0.590
<60 min a day	132	61.4%	28	24.4%		65	59.6%	67	63.2%	
VPA										
≥20 min a day at least 3 days a week	190	88.4%	113	98.3%	<**0.001** ***	95	87.2%	95	89.6%	0.572
<20 min a day at least 3 days a week	25	11.6%	2	1.7%		14	12.8%	11	10.4%	
NUMBER OF STEPS A DAY										
Below norm for age and gender	159	74.0%	64	55.7%	<**0.001** ***	79	72.5%	80	75.5%	0.616
Within norm for age and gender	56	26.1%	51	44.4%		30	27.5%	26	24.5%	

*n*—Number of participants; *p* value—probability level; Z score = result of Mann–Whitney U-Test; bold values—statistically significant; *** *p* < 0.001; bold values—statistically significant.

**Table 4:** Comparison of physical activity rates in the diabetes and the control groups depending on sex.

Physical Activity Rates/Data from 7-Day Study	Girls (*n* = 169)					Boys (*n* = 161)				
	Diabetes Group (*n* = 119)		Control Group (*n* = 50)		*p* Value	Diabetes Group (*n* = 96)		Control Group (*n* = 65)		*p* Value
	Mean	SD	Mean	SD		Mean	SD	Mean	SD	
SEDENTARY [min]	3760.50	1145.57	3598.78	818.40	0.283	3575.98	1152.56	3577.69	949.20	0.884
LIGHT (LPA) [min]	1014.48	356.68	1039.20	322.38	0.616	1085.36	396.76	1068.18	307.66	0.729
MODERATE (MPA) [min]	245.62	160.15	263.12	83.62	**0.007** **	266.20	156.94	272.31	84.20	**0.031** *
VIGOROUS (VPA) [min]	216.74	255.42	212.18	98.55	**0.001** **	251.38	263.41	206.86	106.32	0.069
% SEDENTARY	71.41	12.47	70.11	8.09	**0.030** *	68.87	12.02	69.29	8.21	0.529
% LIGHT (LPA)	19.53	6.14	20.54	6.16	0.244	21.18	6.39	21.12	5.77	0.770
% MODERATE (MPA)	4.79	3.08	5.21	1.61	**0.002** **	5.16	2.75	5.48	1.95	0.051
% VIGOROUS (VPA)	4.27	5.00	4.14	1.92	<**0.001** ***	4.79	4.85	4.12	2.12	0.057
TOTAL MVPA	462.36	405.52	475.30	154.22	<**0.001** ***	517.58	408.55	479.17	163.59	**0.026** *
% MVPA	9.06	7.87	9.35	2.93	<**0.001** ***	9.95	7.35	9.59	3.58	**0.036** *
Mean MVPA/day	72.12	60.99	75.05	23.40	<**0.001** ***	81.16	61.98	77.42	27.23	**0.009** **
Total number of steps/study period	54,629.43	18,168.06	67,028.10	16,692.78	<**0.001** ***	59,616.69	24,022.16	66,609.60	15,998.81	**0.010** *
Mean number of steps/day	8512.42	2553.39	10,651.91	2760.71	<**0.001** ***	9436.77	3665.61	10,766.96	2738.23	**0.002** **
Mean number of steps/minute	10.64	3.40	13.26	3.39	<**0.001** ***	11.75	4.65	13.35	3.78	**0.007** **

*n*—Number of participants; SD—standard deviation; *p* value—probability level; *** *p* < 0.001, ** *p* < 0.01, * *p* < 0.05.

**Table 5:** Physical activity rates in the diabetes and the control groups depending on sex.

**Physical Activity Rates/Data from 7-Day Study**	**Diabetes Group (*n* = 215)**								
	**Girls**	**(*n* = 119)**	**Boys**	**(*n* = 96)**	**Mean Difference (Girls/Boys)**	**−95% CI**	**95% CI**	**t/Z**	***p* Value**
	**Mean**	**SD**	**Mean**	**SD**					
SEDENTARY [min]	3760.5	±1145.6	3576.0	±1152.6	184.5	175.3	193.7	1.42	0.155
LIGHT (LPA) [min]	1014.5	±356.7	1085.4	±396.8	70.9	67.3	74.4	−1.04	0.298
MODERATE (MPA) [min]	245.6	±160.1	266.2	±156.9	20.6	19.5	21.6	−1.50	0.134
VIGOROUS (VPA) [min]	216.7	±255.4	251.4	±263.4	34.6	32.9	36.4	−1.73	0.083
% SEDENTARY	71.4	±12.5	68.9	±12.0	2.5	2.4	2.7	**2.45**	**0.014** *
% LIGHT (LPA)	19.5	±6.1	21.2	±6.4	1.6	1.6	1.7	−**2.09**	**0.036** *
% MODERATE (MPA)	4.8	±3.1	5.2	±2.8	0.4	0.3	0.4	−1.74	0.081
% VIGOROUS (VPA)	4.3	±5.0	4.8	±4.9	0.5	0.5	0.6	−1.93	0.054
TOTAL MVPA	62.4	±405.5	517.6	±408.5	55.2	52.5	58.0	−1.87	0.062
% MVPA	9.1	±7.9	9.9	±7.3	0.9	0.8	0.9	−**2.07**	**0.038** *
Mean MVPA/day	72.1	±61.0	81.2	±62.0	9.0	8.6	9.5	−**2.04**	**0.041** *
**Physical Activity Rates/Data from 7-Day Study**	**Control Group (*n* = 115)**								
	**Girls**	**(*n* = 50)**	**Boys**	**(*n* = 65)**	**Mean Difference (Girls/Boys)**	**−95% CI**	**95% CI**	**t/Z**	***p* Value**
	**Mean**	**SD**	**Mean**	**SD**					
SEDENTARY [min]	3598.8	±818.4	3577.7	±949.2	21.1	20.0	22.1	0.13	0.900
LIGHT (LPA) [min]	1039.2	±322.4	1068.2	±307.7	29.0	27.5	30.4	−0.49	0.624
MODERATE (MPA) [min]	263.1	±83.6	272.3	±84.2	9.2	8.7	9.6	0.10	0.919
VIGOROUS (VPA) [min]	212.2	±98.6	206.9	±106.3	5.3	5.1	5.6	0.78	0.438
% SEDENTARY	70.1	±8.1	69.3	±8.2	0.8	0.8	0.9	0.32	0.750
% LIGHT (LPA)	20.5	±6.2	21.2	±5.8	0.6	0.6	0.6	−0.52	0.602
% MODERATE (MPA)	5.2	±1.6	5.5	±2.0	0.3	0.3	0.3	−0.05	0.957
% VIGOROUS (VPA)	4.1	±1.9	4.1	±2.1	0.0	0.0	0.0	0.17	0.866
TOTAL MVPA	475.3	±154.2	479.2	±163.6	3.9	3.7	4.1	0.32	0.748
% MVPA	9.4	±2.9	9.6	±3.6	0.2	0.2	0.3	0.18	0.859
Mean MVPA/day	75.1	±32.4	77.4	±27.2	2.4	2.3	2.5	0.07	0.944

*n*—Number of participants; Me—median; Z score = result of Mann–Whitney U-Test; CL—confidence interval; SD—standard deviation; *p* value—probability level; *** *p* < 0.001, ** *p* < 0.01, * *p* < 0.05; bold values—statistically significant.

**Table 6:** Comparison of physical activity rates in the diabetes and the control groups depending on age.

Physical Activity Rates/Data from 7-Day Study	6–12 Years (*n* = 154)					13–18 Years (*n* = 66)				
	Diabetes Group (*n* = 91)		Control Group (*n* = 63)		*p* Value	Diabetes Group (*n* = 14)		Control Group (*n* = 52)		*p* Value
	Mean	SD	Mean	SD		Mean	SD	Mean	SD	
SEDENTARY [min]	3542.06	1151.58	3302.19	705.41	0.157	3779.87	1142.36	3908.43	972.36	0.391
LIGHT (LPA) [min]	1165.22	344.39	1191.14	273.13	0.409	957.06	375.05	902.45	285.20	0.610
MODERATE (MPA) [min]	267.94	175.28	293.95	71.66	<**0.001** ***	244.99	145.01	239.36	87.44	0.402
VIGOROUS (VPA) [min]	227.89	292.61	220.42	96.33	<**0.001** ***	235.44	231.86	196.46	108.77	0.247
% SEDENTARY	67.69	13.01	65.57	6.90	**0.001** **	72.21	11.43	74.25	6.89	0.845
% LIGHT (LPA)	22.72	5.78	23.96	4.85	0.076	18.43	6.04	17.37	5.03	0.315
% MODERATE (MPA)	5.20	3.26	5.98	1.62	<**0.001** ***	4.77	2.67	4.66	1.77	0.440
% VIGOROUS (VPA)	4.39	5.57	4.49	2.10	<**0.001** ***	4.59	4.41	3.72	1.87	0.198
TOTAL MVPA	495.83	461.15	514.37	149.32	<**0.001** ***	480.43	362.81	435.82	160.42	0.218
% MVPA	9.59	8.70	10.47	3.34	<**0.001** ***	9.36	6.78	8.38	2.90	0.208
Mean MVPA/day	75.64	67.33	82.52	25.48	<**0.001** ***	76.54	56.95	69.46	24.02	0.083
Total number of steps /study period	57,085.41	20,241.38	69,872.77	10,012.47	<**0.001** ***	56,684.93	21,766.19	63,310.93	20,737.36	**0.044** *
Mean number of steps/day	8844.87	2926.86	11,265.12	2254.33	<**0.001** ***	8985.21	3277.64	10,097.70	3101.23	**0.017** *
Mean number of steps/minute	11.23	3.97	14.26	2.93	<**0.001** ***	11.06	4.10	12.24	4.00	0.082

*n*—Number of participants; SD—standard deviation; *p* value—probability level; *** *p* < 0.001, ** *p* < 0.01, * *p* < 0.05.

**Table 7:** Physical activity rates in the diabetes and the control groups depending on age.

**Physical Activity Rates/Data from 7-Day Study**	**Diabetes Group (*n* = 215)**								
	**6–12 Years**	**(*n* = 91)**	**13–18 Years**	**(*n* = 14)**	**Mean Difference (6–12/13–18)**	**−95% CI**	**95% CI**	**t/Z**	***p* Value**
	**Mean**	**SD**	**Mean**	**SD**					
SEDENTARY [min]	3542.1	±1151.6	3779.9	1142.4	237.8	225.9	249.7	−1.49	0.135
LIGHT (LPA) [min]	1165.2	±344.4	957.1	375.0	208.2	197.7	218.6	**4.30**	<**0.001** ***
MODERATE (MPA) [min]	267.9	±175.3	245.0	145.0	23.0	21.8	24.1	0.69	0.487
VIGOROUS (VPA) [min]	227.9	±292.6	235.4	231.9	7.6	7.2	7.9	−0.69	0.336
% SEDENTARY	67.7	±13.0	72.2	11.4	4.5	4.3	4.7	−**3.49**	<**0.001** ***
% LIGHT (LPA)	22.7	±5.8	18.4	6.0	4.3	4.1	4.5	**5.44**	<**0.001** ***
% MODERATE (MPA)	5.2	±3.3	4.8	2.7	0.4	0.4	0.5	0.97	0.332
% VIGORIOUS (VPA)	4.4	±5.6	4.6	4.4	0.2	0.2	0.2	−0.65	0.515
TOTAL MVPA	495.8	±461.2	480.4	362.8	15.4	14.6	16.2	−0.39	0.699
% MVPA	9.6	±8.7	9.4	6.8	0.2	0.2	0.2	−0.22	0.828
Mean MVPA/day	75.6	±67.3	76.5	56.9	0.9	0.9	0.9	−0.47	0.638
**Physical Activity Rates/Data from 7-Day Study**	**Control Group (*n* = 115)**								
	**6–12 Years**	**(*n* = 63)**	**13–18 Years**	**(*n* = 52)**	**Mean Difference (6** **−** **12/13** **−** **18)**	**−95% CI**	**95% CI**	**t/Z**	***p* Value**
	**Mean**	**SD**	**Mean**	**SD**					
SEDENTARY [min]	3302.2	±705.4	3908.4	±972.4	606.2	575.9	636.6	−**3.48**	**0.001** **
LIGHT (LPA) [min]	1191.1	±273.1	902.5	±285.2	288.7	274.3	303.1	**4.96**	<**0.001** ***
MODERATE (MPA) [min]	294.0	±71.7	239.4	±87.4	54.6	51.9	57.3	**3.31**	**0.001** **
VIGOROUS (VPA) [min]	220.4	±96.3	196.5	±108.8	24.0	22.8	25.2	**2.03**	**0.042** *
% SEDENTARY	65.6	±6.9	74.2	±6.9	8.7	8.2	9.1	−**5.88**	<**0.001** ***
% LIGHT (LPA)	24.0	±4.9	17.4	±5.0	6.6	6.3	6.9	**6.11**	<**0.001** ***
% MODERATE (MPA)	6.0	±1.6	4.7	±1.8	1.3	1.3	1.4	**4.16**	<**0.001** ***
% VIGORIOUS (VPA)	4.5	±2.1	3.7	±1.9	0.8	0.7	0.8	**2.54**	**0.011** *
TOTAL MVPA	514.4	±149.3	435.8	±160.4	78.6	74.6	82.5	**2.89**	**0.004** **
% MVPA	10.5	±3.3	8.4	±2.9	2.1	2.0	2.2	**3.84**	<**0.001** ***
Mean MVPA/day	82.5	±25.5	69.5	±24.0	13.1	12.4	13.7	**3.00**	**0.003** **

*n*—Number of participants; Me—median; Z score = result of Mann–Whitney U-Test; CI—confidence interval; SD—standard deviation; *p* value—probability level; *** *p* < 0.001, ** *p* < 0.01, * *p* < 0.05; bold values—statistically significant.

**Table 8:** Comparison of physical activity rates in the diabetes and the control groups depending on sex and age.

**Physical Activity Rates/Data from 7-Day Study**	**Girls 6–12 Years (*n* = 73)**					**Girls 13–18 Years (*n* = 96)**				
	**Diabetes Group (*n* = 48)**		**Control Group (*n* = 25)**		***p* Value**	**Diabetes Group (*n* = 71)**		**Control Group (*n* = 25)**		***p* Value**
	**Mean**	**SD**	**Mean**	**SD**		**Mean**	**SD**	**Mean**	**SD**	
SEDENTARY [min]	3673.5	1204.8	3352.8	740.7	0.161	3819.3	1108.6	3844.8	832.3	0.933
LIGHT (LPA) [min]	1163.0	306.5	1203.4	273.5	0.526	914.0	355.0	875.0	285.0	0.854
MODERATE (MPA) [min]	260.1	181.5	297.1	62.7	**0.001** **	235.9	144.5	229.2	89.1	0.670
VIGOROUS (VPA) [min]	219.8	296.9	225.6	83.0	<**0.001** ***	214.6	225.3	198.8	112.1	0.184
% SEDENTARY	68.4	13.3	65.7	6.1	**0.011** *	73.4	11.5	74.5	7.5	0.713
% LIGHT (LPA)	22.3	5.8	23.9	4.9	0.224	17.6	5.6	17.2	5.4	0.742
% MODERATE (MPA)	5.0	3.4	5.9	1.1	<**0.001** ***	4.6	2.8	4.5	1.7	0.567
% VIGORIOUS (VPA)	4.2	5.6	4.5	1.7	<**0.001** ***	4.3	4.6	3.8	2.1	0.104
TOTAL MVPA	479.9	473.7	522.6	120.6	<**0.001** ***	450.5	355.2	428.0	171.3	0.214
% MVPA	9.2	8.9	10.4	2.3	<**0.001** ***	8.9	7.1	8.3	3.2	0.193
Mean MVPA/day	71.6	65.6	83.5	17.6	<**0.001** ***	72.5	58.2	66.6	25.7	0.149
Total number of steps/study period	56,335.2	18,286.6	70,311.7	10,965.0	<**0.001** ***	53,476.2	18,125.9	63,744.5	20,644.9	**0.031** *
Mean number of steps/day	8597.2	2753.0	11,357.4	2247.9	<**0.001** ***	8455.1	2427.7	9946.5	3077.5	**0.033** *
Mean number of steps/minute	10.9	3.8	14.1	2.7	<**0.001** ***	10.4	3.1	12.4	3.8	**0.039** *
**Physical Activity Rates/Data from 7-Day Study**	**Boys 6–12 Years (*n* = 81)**					**Boys 13–18 Years (*n* = 80)**				
	**Diabetes Group (*n* = 45)**		**Control Group (*n* = 36)**		***p* Value**	**Diabetes Group (*n* = 51)**		**Control Group (*n* = 29)**		***p* Value**
	**Mean**	**SD**	**Mean**	**SD**		**Mean**	**SD**	**Mean**	**SD**	
SEDENTARY [min]	3398.7	1086.1	3267.1	688.3	0.753	3726.0	1195.7	3963.3	1090.5	0.247
LIGHT (LPA) [min]	1167.6	385.1	1182.6	276.4	0.639	1015.8	396.8	926.1	288.2	0.478
MODERATE (MPA) [min]	276.6	169.9	291.8	78.1	**0.009** *	257.4	146.2	248.1	86.5	0.741
VIGOROUS (VPA) [min]	236.7	291.0	216.8	105.6	**0.003** **	263.8	239.8	194.5	107.7	0.871
% SEDENTARY	66.9	12.8	65.5	7.5	**0.028** *	70.5	11.2	74.0	6.5	0.243
% LIGHT (LPA)	23.1	5.8	24.0	4.9	0.124	19.5	6.5	17.5	4.7	0.126
% MODERATE (MPA)	5.4	3.1	6.0	1.9	**0.007** **	4.9	2.4	4.8	1.8	0.855
% VIGORIOUS (VPA)	4.6	5.6	4.5	2.4	**0.001** **	5.0	4.2	3.7	1.7	0.783
TOTAL MVPA	513.2	451.9	508.6	167.8	**0.002** **	521.3	372.4	442.6	153.2	0.886
% MVPA	10.0	8.5	10.5	3.9	**0.001** **	9.9	6.3	8.4	2.7	0.933
Mean MVPA/day	80.0	69.7	81.9	30.0	**0.001** **	82.1	55.3	71.9	22.7	0.668
Total number of steps/study period	57,903.8	22,366.2	69,567.9	9443.4	**0.007** **	61,066.1	25,465.1	62,937.2	21,174.6	0.626
Mean number of steps/day	9115.0	3114.8	11,201.1	2288.4	**0.001** **	9709.0	4084.6	10,228.1	3170.0	0.394
Mean number of steps/minute	11.6	4.1	14.4	3.1	**0.001** **	11.9	5.1	12.1	4.2	0.871

*n*—Number of participants; SD—standard deviation; *p* value—probability level; *** *p* < 0.001, ** *p* < 0.01, * *p* < 0.05.

**Table 9:** Physical activity rates in the diabetes and the control groups depending on sex and age.

**Physical Activity Rates/Data from 7-Day Study**	**Diabetes Group (*n* = 215)**										
	**Girls 6–12 Years (*n* = 48)**		**Boys 6–12 Years (*n* = 45)**		**Girls 13–18 Years (*n* = 71)**		**Boys 13–18 Years (*n* = 51)**		***p* Value**		
	**Mean**	**SD**	**Mean**	**SD**	**Mean**	**SD**	**Mean**	**SD**	***p* Sex**	***p* Age**	***p* Sex × Age**
SEDENTARY [min]	3673.5	±1204.8	3398.7	±1086.1	3819.3	±1108.6	3726.0	±1195.7	0.308	<**0.001** ***	0.404
LIGHT (LPA) [min]	1163.0	±306.5	1167.6	±385.1	914.0	±355.0	1015.8	±396.8	0.248	<**0.001** ***	0.433
MODERATE [min]	260.1	±181.5	276.6	±169.9	235.9	±144.5	257.4	±146.2	0.369	0.296	0.976
VIGOROUS [min]	219.8	±296.9	236.7	±291.0	214.6	±225.3	263.8	±239.8	0.365	0.883	0.670
% SEDENTARY	68.4	±13.3	66.9	±12.8	73.4	±11.5	70.5	±11.2	0.172	**0.012** **	0.716
% LIGHT (LPA)	22.3	±5.8	23.1	±5.8	17.6	±5.6	19.5	±6.5	0.083	<**0.001** ***	0.529
% MODERATE (MPA)	5.0	±3.4	5.4	±3.1	4.6	±2.8	4.9	±2.4	0.373	0.305	0.809
% VIGORIOUS (VPA)	4.2	±5.6	4.6	±5.6	4.3	±4.6	5.0	±4.2	0.460	0.827	0.781
TOTAL MVPA	479.9	±473.7	513.2	±451.9	450.5	±355.2	521.3	±372.4	0.354	0.753	0.795
% MVPA	9.2	±8.9	10.0	±8.5	8.9	±7.1	9.9	±6.3	0.414	0.800	0.930
Mean MVPA/ day	71.6	±65.6	80.0	±69.7	72.5	±58.2	82.1	±55.3	0.293	0.956	0.967
**Physical Activity Rates/Data from 7-Day Study**	**Control Group (*n* = 115)**										
	**Girls 6–12 Years (*n* = 25)**		**Boys 6–12 Years (*n* = 36)**		**Girls 13–18 Years (*n* = 25)**		**Boys 13–18 Years (*n* = 29)**		***p* Value**		
	**Mean**	**SD**	**Mean**	**SD**	**Mean**	**SD**	**Mean**	**SD**	***p* Sex**	***p* Age**	***p* Sex × Age**
SEDENTARY [min]	3352.8	±740.7	3267.1	±688.3	3844.8	±832.3	3963.3	±1090.5	0.805	<**0.001** ***	0.548
LIGHT (LPA) [min]	1203.4	±273.5	1182.6	±276.4	875.0	±285.0	926.1	±288.2	0.861	<**0.001** ***	0.572
MODERATE [min]	297.1	±62.7	291.8	±78.1	229.2	±89.1	248.1	±86.5	0.699	<**0.001** ***	0.254
VIGOROUS [min]	225.6	±83.0	216.8	±105.6	198.8	±112.1	194.5	±107.7	0.782	0.295	0.321
% SEDENTARY	65.7	±6.1	65.5	±7.5	74.5	±7.5	74.0	±6.5	0.901	<**0.001** ***	0.740
% LIGHT (LPA)	23.9	±4.9	24.0	±4.9	17.2	±5.4	17.5	±4.7	0.957	<**0.001** ***	0.925
% MODERATE (MPA)	5.9	±1.1	6.0	±1.9	4.5	±1.7	4.8	±1.8	0.598	<**0.001** ***	0.589
% VIGORIOUS (VPA)	4.5	±1.7	4.5	±2.4	3.8	±2.1	3.7	±1.7	0.884	**0.042** *	0.403
TOTAL MVPA	522.6	±120.6	508.6	±167.8	428.0	±171.3	442.6	±153.2	0.992	**0.003** **	0.217
% MVPA	10.4	±2.3	10.5	±3.9	8.3	±3.2	8.4	±2.7	0.857	<**0.001** ***	0.409
Mean MVPA/ day	83.5	±17.6	81.9	±30.0	66.6	±25.7	71.9	±22.7	0.696	**0.001** **	0.094

*n*—Number of participants; SD—standard deviation; *p* value—probability level; *** *p* < 0.001, ** *p* < 0.01, * *p* < 0.05; bold values—statistically significant.

## Abbreviations

T1D	Type one diabetes;
PE	physical education;
MODY	Maturity Onset Diabetes of the Young
MDI	multiple daily injections;
CSII	continuous subcutaneous insulin infusion;
LPA	light physical activity;
MPA	moderate physical activity;
VPA	vigorous physical activity;
BMI	body mass index
HbA1C	glycated haemoglobin
MVPA	moderate to vigorous physical activity;
cpm	count per minutes;
CGM	continuous glucose monitoring;
rtCGM	real-time continuous glucose monitoring;
iCGM	intermittently continuous glucose monitoring.
